# Autoimmune Cytopenias Post Hematopoietic Stem Cell Transplantation in Pediatric Patients With Osteopetrosis and Other Nonmalignant Diseases

**DOI:** 10.3389/fimmu.2022.879994

**Published:** 2022-05-27

**Authors:** Ehud Even-Or, Yael Dinur Schejter, Adeeb NaserEddin, Irina Zaidman, Bella Shadur, Polina Stepensky

**Affiliations:** ^1^ Department of Bone Marrow Transplantation and Cancer Immunotherapy, Hadassah Medical Center, Faculty of Medicine, Hebrew University of Jerusalem, Jerusalem, Israel; ^2^ Immunology Division, The Garvan Institute of Medical Research Graduate Research School, University of New South Wales, Sydney, NSW, Australia

**Keywords:** autoimmune cytopenia, hematopoietic stem cell transplantation, osteopetrosis, nonmalignant, immune thrombocytopenia, autoimmune hemolytic anemia, autoimmune neutropenia, pediatrics

## Abstract

Autoimmune cytopenia (AIC) is a rare complication post hematopoietic stem cell transplantation (HSCT), with a higher incidence in nonmalignant diseases. The etiology of post-HSCT AIC is poorly understood, and in many cases, the cytopenia is prolonged and refractory to treatment. Diagnosis of post-HSCT AIC may be challenging, and there is no consensus for a standard of care. In this retrospective study, we summarize our experience over the past five years with post-HSCT AIC in pediatric patients with osteopetrosis and other nonmalignant diseases. All pediatric patients who underwent HSCT for nonmalignant diseases at Hadassah Medical Center over the past five years were screened for post-HSCT AIC, and data were collected from the patient’s medical records. From January 2017 through December 2021, 140 pediatric patients underwent HSCT for osteopetrosis (n=40), and a variety of other nonmalignant diseases. Thirteen patients (9.3%) presented with post-HSCT AIC. Of these, 7 had osteopetrosis (17.5%), and 6 had other underlying nonmalignant diseases. Factors associated with developing AIC included unrelated or non-sibling family donors (n=10), mixed chimerism (n=6), and chronic GvHD (n=5). Treatment modalities included steroids, IVIG, rituximab, bortezomib, daratumumab, eltrombopag, plasmapheresis, and repeated HSCT. Response to treatment was variable; Seven patients (54%) recovered completely, and three patients (23%) recovered partially, still suffering from mild-moderate thrombocytopenia. Three patients died (23%), two following progressive lung disease and one from sepsis and multi-organ failure after a 3^rd^ HSCT. In our experience, post-HSCT AICs in pediatric patients with nonmalignant diseases may pose a challenging post-transplant complication with a variable presentation and a wide spectrum of severity. A relatively high prevalence is seen in patients with osteopetrosis, possibly due to difficult engraftment and high rates of mixed chimerism. There is a dire need for novel treatment modalities for better management of the more severe and refractory cases.

## Introduction

Allogeneic hematopoietic stem cell transplantation (HSCT) is a curative treatment for various pediatric nonmalignant diseases including bone marrow failures, hemoglobinopathies, immune deficiencies, and inborn errors of metabolism (1). With constant improvement in supportive care and the introduction of novel treatments, outcomes of these transplants are improving and Indications for HSCT in pediatric nonmalignant diseases are constantly expanding (2).

Autoimmune cytopenia (AIC) post**-**HSCT is a relatively rare complication, with reported incidence ranging between 2.5-5% in pediatric patients. However, the incidence is much higher in patients undergoing HSCT for nonmalignant diseases, reaching 20-35% (3). The etiology of post-HSCT AIC is poorly understood and involves immune dysregulation and imbalance between autoreactive and autoregulatory lymphocytes during the process of post-HSCT immune reconstitution (4, 5). The differential diagnosis of post-HSCT AIC is broad, including viral infections, graft versus host disease (GvHD) related cytopenia, and drug toxicity causing myelosuppression. In many cases, the course of these complications is prolonged and often they are refractory to treatment, with high rates of morbidity and mortality. Therefore, diagnosis and management of post-HSCT AIC are often challenging, and there is no well-established standard of care (3–9).

In this study, we summarize our experience over the past five years with post-HSCT AIC in pediatric patients with nonmalignant diseases.

## Methods

### Patients

In this retrospective study, all pediatric patients who underwent HSCT for nonmalignant diseases at Hadassah-Hebrew University Medical Center between January 2017 and December 2021 were screened for post-HSCT AICs. Criteria for inclusion in the AIC cohort included an AIC in any one of the three hematopoietic cell lines post-HSCT. Diagnosis of AIHA was established by a positive direct Coombs test and serum markers of hemolysis including elevated reticulocyte counts, low serum haptoglobin, and elevated serum LDH. Diagnosis of immune thrombocytopenia and autoimmune neutropenia was established by the exclusion of other causes for the cytopenia post HSCT such as drug toxicity, GvHD, or an underlying viral infection. Bone marrow biopsy was done in relevant cases to assess marrow cellularity and rule out cytopenia due to marrow insufficiency.

### Data Collection

Data were collected from the patient’s medical charts and included demographic, clinical, and transplant-related data. Clinical data included the underlying disease and indication for HSCT, time of appearance of AIC, type of AIC, treatment modalities, response to treatment, other post-HSCT complications, and outcomes. Transplant-related data included graft source and degree of match, conditioning regimen, GvHD prophylaxis, chimerism status at the time of AIC onset as per STR test, GvHD sites, severity, and management if applicable. “Full-donor” was defined as an STR test of 100% donor cells in the recipients’ blood, “mixed chimerism” was defined as any percentage of donor cells in the recipients’ blood below 100% and above 0%, and “recipient” chimerism was defined as 0% donor cells. This study was approved by our institutional Helsinki review board.

### Statistical Analysis

Categorical variables were summarized as number and percentage, and continuous variables as median and range. Clinical and transplant characteristics were compared between patients who developed AIC and those who did not. Fisher’s exact test was used to determine the relationship between categorical independent variables and the development of AIC. Comparison of continuous variables between patients with and without AIC was done by a T-test. A p-value of <0.05 was considered statistically significant. The statistical analysis was done using the software R version 4.1.2.

## Results

From January 2017 through December 2021, 140 pediatric patients underwent HSCT for a variety of nonmalignant diseases. Of these, thirteen patients (9.3%) presented with post-HSCT AIC. The clinical and transplant characteristics of screened patients, with a comparison between those who developed AIC and those who did not, are depicted in **Table 1**. No statistically significant differences were found between the AIC and the no-AIC groups in the clinical and transplant-related variables that were compared. The average age of the patients in the AIC cohort at the time of HSCT was 6.6 years, ranging from 5 months to 17.8 years. **Table 2** depicts the clinical and transplant characteristics of the 13 patients who were included in the AIC cohort.

**Table 1:** Clinical and transplant-related characteristics of all the pediatric patients with nonmalignant diseases who underwent HSCT during the study period at Hadassah Medical Center, with a comparison between the AIC and no-AIC groups.

		AIC	No AIC	All patients	P-value
**Total**		13 (9.3%)	127 (90.7%)	140	
**Gender**	(males, females)	9, 4	76, 51	85, 55	0.5673
**Age at HSCT**	(years)	6.6 (0.4-17.8)	5.2 (0.1-17.8)	5.4 (0.1-17.8)	0.4782
**Disease**	Osteopetrosis	7 (54%)	33 (26%)	40 (29%)	0.125
	Bone marrow failures	3 (23%)	33 (26%)	36 (26%)	
	Immune deficiencies	2 (15%)	49 (39%)	51 (36%)	
	Metabolic diseases	0	11 (9%)	11 (8%)	
	Other	1 (8%)	1 (0.8%)	2 (1.4%)	
**Donor**	Matched sibling	3 (23%)	45 (35%)	48 (34%)	0.2387
	Other matched family	2 (15%)	8 (63%)	10 (7%)	
	Haploidentical	0	7 (6%)	7 (5%)	
	Matched unrelated	3 (23%)	42 (33%)	45 (32%)	
	Mismatched unrelated (9/10)	5 (38%)	25 (20%)	30 (21%)	
**Graft source**	Bone marrow	9 (69%)	103 (81%)	112 (80%)	0.3972
	Peripheral blood	4 (31%)	22 (17%)	26 (19%)	
	Cord blood	0	2 (1.6%)	2 (1.4%)	
**Chimerism**	Full donor	7 (54%)	93 (73%)	100 (71%)	0.2665
	Mixed chimera	6 (46%)	30 (24%)	36 (26%)	
	Recipient		2 (1.6%)	2 (1.4%)	
**GvHD**	none	7 (54%)	76 (60%)	83 (59%)	0.1042
	grade I/II	4 (31%)	14 (11%)	18 (13%)	
	grade III/IV	2 (15%)	34 (27%)	36 (26%)	
**2nd transplant**		2 (15%)	10 (8%)	12 (8.6%)	0.3082
**Mortality**		3 (23%)	14 (11%)	17 (12%)	0.22

**Table 2:** AIC cohort - patient and transplant characteristics.

Pt #	Primary disease	Age atTransplant(years)	Donor	HLA match	Graftsource	Conditioningregimen	GvHDprophylaxis
**1**	Osteopetrosis	7.3	grandmother	10/10	BM	Flu Treo TT ATG	CSA + MMF
**2**	Osteopetrosis	0.4	father	9/10	BM	Flu Treo TT ATG	CSA + MMF
**3**	Glanzmann Thrombasthenia	13.8	brother	10/10	BM	Rtx Cmp Bu Flu	CSA + MMF
**4**	VPS45 deficiency	0.8	unrelated	9/10	PBSC	Bu Flu TT ATG	CSA + MMF
**5**	Dyskeratosis Congenita	4.4	unrelated	10/10	BM	Cmp Flu Mel	CSA + MMF
			different unrelated	10/10	PBSC	Flu Treo ATG	CSA + MMF
**6**	Osteopetrosis	5.1	unrelated	9/10	BM	Flu Treo TT ATG	CSA + MMF
**7**	Osteopetrosis	1.4	unrelated	10/10	BM	Flu Treo TT ATG	CSA + MMF
**8**	Osteopetrosis	0.8	unrelated	9/10	BM	Flu Treo TT ATG	CSA + MMF
**9**	XLP1	14.9	unrelated	10/10	BM	Bu Flu ATG	CSA + MTX
**10**	Severe aplastic anemia	17.8	sister	10/10	BM	Flu Cy ATG	CSA + MMF
**11**	Severe aplastic anemia	16.4	sister	10/10	BM	Flu Cy ATG	CSA + MMF
			same sister	10/10	PBSC	Cmp Flu Cy TBI	CSA + MTX
**12**	Osteopetrosis	1.3	unrelated	9/10	PBSC	Flu Treo TT ATG	CSA + MTX
**13**	Osteopetrosis	1.1	unrelated	9/10	BM	Flu Treo TT ATG	CSA + MMF

HLA, human leukocyte antigen; GvHD, graft-versus-host disease; BM; bone marrow; PBSC, peripheral blood stem cells; Flu, fludarabine;Treo, treosulfan; TT ,thiotepa; ATG, anti-thymocyte globulins; Rtx, rituximab; Cmp, alemtuzumab (campath); Bu, busulfan;Cy, cyclophosphamide; TBI, total body irradiation; CSA, cyclosporin; MMF, mycophenolate mofetil; MTX, methotrexate;XLP1, x-linked lymphoproliferative disease 1.

### Underlying Disease

Osteopetrosis is the most common nonmalignant underlying disease in our cohort. Out of all 140 patients who were screened for AIC, 40 had osteopetrosis, and out of the 13 patients who developed AIC, 7 (54%) had osteopetrosis. The prevalence of post-HSCT AIC in osteopetrosis patients in our cohort is 17.5%. Other underlying diseases in the AIC cohort include bone marrow failures (n=3), immune deficiencies (n=2), and Glanzmann thrombasthenia (n=1). Of note, in two patients (patients #5,11), the AIC developed following a second transplant.

### Donor and Graft Source

In the AIC cohort, only 3 of the 13 patients were transplanted from HLA-matched sibling donors. The other 10 patients were transplanted from alternative donors: one from a one-allele mismatched father, one from his HLA-matched grandmother, 3 from matched unrelated donors, and 5 from one-allele mismatched unrelated donors. The graft source was bone marrow in 9 patients and peripheral blood stem cells (PBSC) in the other 4 patients.

### Conditioning Regimen

The conditioning regimen was myeloablative in eleven of the thirteen patients of the AIC cohort; three of the patients received a busulfan-based regimen and eight patients received a reduced-toxicity, treosulfan-based regimen. Two patients, who were transplanted for severe aplastic anemia from matched siblings, received a reduced-intensity regimen with fludarabine and cyclophosphamide (120 mg/kg). One of them (patient #11) was a second transplant following secondary graft failure, and to enhance engraftment, he received a PBSC-collected graft, alemtuzumab for more intensive pre-HSCT lymphodepletion, and low-dose total body irradiation (3 Gy). All patients received serotherapy (either ATG or alemtuzumab), and cyclosporine with either mycophenolate mofetil or methotrexate for GvHD prevention.

### AIC Characteristics

The AIC characteristics, various treatments, and outcomes are summarized in **Table 3**. The median time from HSCT to AIC onset was 74 days, ranging from the time of engraftment to one-year post-HSCT. In patients #5 and #11, the days to AIC onset were counted starting from their 2^nd^ HSCT. Types of cytopenia were variable and included: isolated thrombocytopenia (n=3), isolated hemolytic anemia (n=1), bi-cytopenia (n=4), and tri-lineage pan-cytopenia (n=5).

**Table 3:** AICs, treatments and outcomes.

Pt #	Time from HSCT to AIC initial symptoms (days)	Medications at time of initial AIC symptoms	AIHA	ITP	AIN	Duration of cytopenia	Treatments for AIC (duration/doses)	Response to treatment	Chimerism in STR test during AIC	GvHD	Outcome
**1**	55	CSA	**√**	**√**	**√**	10 days	steroids (4 weeks), IVIG (2 doses)	CR	95-98%	no	alive and well
**2**	356	none	**√**	**√**		ongoing	steroids (2.5 months), eltrombopag (1 year), rituximab (3 doses), 2nd HSCT	CR	56-26%	severe GI	alive and well
**3**	since HSCT	CSA		**√**		ongoing	rituximab (4 doses), eltrombopag (3.5 years)	PR	84%-full	no	mild-moderate thrombocytopenia
**4**	103	CSA	**√**	**√**		7 months	steroids (8.5 months), rapamycin (2 months), rituximab (7 doses), abatacept (4 doses), bortezomib (5 doses), daratumumab (6 doses)	CR	full donor	no	alive and well
**5**	77	CSA, prednisone	**√**	**√**	**√**	20 months	steroids (6 months), rapamycin (3 months), rituximab (4 doses), bortezomib (5 doses), daratumumab (4 doses), 3rd HSCT	No R	full donor	mild skin and GI	deceased
**6**	103	none	**√**			2 months	steroids (2.5 months), IVIG (1 dose), rituximab (4 doses), daratumumab (5 doses), plasma-pheresis (2 times)	CR	14-33%	no	alive and well
**7**	74	none	**√**	**√**	**√**	1 month	no treatments	CR	full-> 45% -> full donor	mild skin and GI	alive and well
**8**	39	CSA		**√**	**√**	ongoing	steroids (4 weeks), rituximab (4 doses), daratumumab (6 doses), eltrombopag (6 months)	PR	full donor	no	moderate thrombocytopenia
**9**	280	Jakavi		**√**		1 month	steroids (2 weeks), IVIG (3 doses)	CR	full donor	chronic skin, eyes, GI	chronic GvHD
**10**	since HSCT	CSA	**√**	**√**	**√**	3 months	steroids (4 weeks)	No R	full donor	no	deceased
**11**	since HSCT	CSA	**√**	**√**	**√**	5 months	steroids (2 months)	No R	full donor	mild GI	deceased
**12**	99	CSA	**√**	**√**		1.5 months	steroids (5 weeks)	CR	24-52%	mild skin	alive and well
**13**	since HSCT	CSA		**√**		ongoing	steroids (3 weeks), rituximab (4 doses)	PR	full donor	no	moderate thrombocytopenia

AIC, autoimmune cytopenia; HSCT, hematopoietic stem cell transplantation; AIHA, autoimmune hemolytic anemia; ITP, immune thrombocytopenia; AIN, autoimmune neutropenia; STR, short tandem repeats; GvHD, graft-versus-host disease; CSA, cyclosporin; IVIG, intra-venous immunoglobulins; MSC, mesenchymal stem cells; GI, gastrointestinal; EBV, Epstein-Barr virus; PTLD, post-transplant lymphoproliferative disease. CR, Complete response; defined cases where complete resolution of the cytopenia was observed. PR, partial response; defined cases where partial improvement in cytopenia was achieved, without a need for blood-product support. No R, no response; defined cases refractory to treatment in which patients continued to require blood-product support. Since HSCT = cytopenias which presented prior to engraftment and persisted as AIC. "√", indicates involvement of the specific cytopenia in the clinical presentation of the patient.

### Chimerism and GvHD

Six of the 13 patients had mixed chimerism and 7 patients had full-donor chimerism on STR tests during the time of AIC. Five of the patients had GvHD at the time of AIC, in four of them mild skin and GI GvHD, and in one patient (patient #9) extensive chronic GvHD of the GI tract, skin, and soft tissues. Another patient (patient #2) had severe GI GvHD which had already resolved at the time of AIC.

### Treatments and Outcomes

In most cases, the first treatment line was steroids (n=10). Other treatment modalities included IVIG (n=3), rituximab (n=7), bortezomib (n=2), daratumumab (n= 4), eltrombopag (n=3), plasmapheresis (n=1), and repeated HSCT (n=2).

Seven patients (54%) completely recovered, three patients (23%) recovered partially and still have mild-moderate thrombocytopenia not requiring platelet transfusions, and three patients (23%) died. Of the deceased patients, one patient (patient #5) died three weeks after a 3^rd^ HSCT due to sepsis and multi-organ failure, and two patients (patients #10,11) died from progressive lung disease concurrent with AIC.

## Discussion

### Incidence Rates

Post-HSCT AICs may pose a challenging post-transplant complication (3). In this study we present our experience over the past 5 years with treating post-HSCT AIC in a cohort of 13 pediatric patients with nonmalignant diseases. These patients were identified and selected out of a total of 140 pediatric patients who underwent HSCT for a variety of nonmalignant diseases, with an incidence rate of 9.3% for developing post-HSCT AIC. This incidence rate in nonmalignant diseases compares well with recent literature. In a study from the Netherlands by Kruizinga et al. 26 patients with AIC were identified out of 531 post-HSCT pediatric patients, 22 of which had nonmalignant diseases, showing an incidence rate of 9.5% for AIC in nonmalignant diseases (5). In another study from California by Neely et al. 20 patients with AIC were identified out of 442 pediatric patients, out of which 9 had primary immune deficiencies (6% incidence), and 4 had other nonmalignant diseases (5.2% incidence) (6). A recent study by Lum et al. showed a cumulative incidence of 9.4% for developing post-HSCT AIC in patients with primary immunodeficiencies (9). And finally, a recent study by Galvin et al. described a cohort of 50 patients with post-HSCT AIC identified out of a cohort of 271 pediatric patients with nonmalignant diseases, showing a cumulative incidence of 18% (8).

The relatively high incidence rate of post-HSCT AIC observed in patients with nonmalignant diseases may be attributed to the chemotherapy-naive and relatively intact pre-HSCT hematopoietic system in these patients, which may increase the risk for persistence of auto-antibody secreting host plasma cells. Also, a more aggressive anti-GvHD approach in nonmalignant patients, including more pre-HSCT serotherapy and increased immunosuppressive treatments, may delay and skew the post-HSCT immune reconstitution process and increase the risk for developing AIC in these patients (10).

### Malignant Infantile Osteopetrosis

Osteopetrosis is a group of rare genetic bone disorders characterized by excessive bone growth due to reduced osteoclast bone resorption (11). The severe, infantile, autosomal recessive disease, often named malignant infantile osteopetrosis (MIOP), is in most cases treatable by HSCT (12). These patients often come to transplant with severely damaged bone marrow due to overgrowing bone into the bone marrow niches and HSCT for these patients is often challenging. Due to high consanguinity rates in some populations in our region and the founder effect, many of our nonmalignant patients have MIOP and our center has gained vast experience with HSCT for treating this condition (13). Out of the 140 patients that were screened in this study, 40 patients had MIOP, of which 7 developed post-HSCT AIC (incidence of 17.5%). The relatively high incidence of AIC in these patients may be attributed to the difficult engraftment and high incidence of mixed chimerism in these transplants. The conditioning regimen we give for MIOP patients includes treosulfan, fludarabine, thiotepa, and ATG. This reduced-toxicity regimen may be less myeloablative than busulfan-based regimens, with relatively high rates of post-HSCT mixed chimerism in osteopetrosis patients (around 35% in our experience), yet highly immunosuppressive, with a significant impact on post-HSCT immune reconstitution (13). This combination of partial engraftment and intense immune suppression may set the stage for developing AIC in these patients.

### Associated Factors

Ten of the thirteen patients in our cohort were transplanted from donors which were either unrelated or non-sibling family donors (**Table 2**). Of the three patients who were transplanted from matched-sibling donors, one was a patient with Glanzmann Thrombasthenia, who was treated with multiple blood products pre-HSCT and thus came to transplant with a hyper-sensitized immune system, and another was a patient with severe aplastic anemia, post 2^nd^ PBSC-collected HSCT from his matched sister, following graft failure.

The high prevalence of alternative, non-matched sibling donors in our cohort compares well with other studies that have defined patients who were transplanted from unrelated donors as a risk group for developing AIC. Additionally, six of the patients in our cohort (46%) were mixed chimeras at the time of AIC onset, suggesting a role of residual antibody-secreting host cells in the pathogenesis, and the lack of myeloablation as a risk factor in the development of AIC (3–8).

Five of the patients (38%) in our cohort had manifestations of GvHD during the AIC. While GvHD is an alloimmune phenomenon involving donor immune recognition of host cells, AIC is thought to be an autoimmune process, involving donor immune recognition of donor-origin hematopoietic cells. However, the pathophysiology in both of these post-HSCT immune complications shares similar elements, both involving impaired immune reconstitution and immune dysregulation. In both, the impaired peripheral tolerance may be attributed to a lack of functional T regulatory (Treg) cells (3). Therefore, an association between GvHD and AIC is plausible.

Unfortunately, due to our relatively small sample size, we have failed to demonstrate statistically significant associations between these factors and post-HSCT AIC.

### Treatment Options

Corticosteroids are usually the first line of treatment for AIC and almost all patients in our cohort were initially treated with either intra-venous methyl-prednisone or oral prednisolone. IVIG, also frequently given as initial treatment, was given to only three patients in our cohort (**Table 3**). In non-responders to first-line treatment, targeting antibody-secreting B-cells with rituximab is usually the next step. Although the response rate to rituximab reaches 60-80% in the literature (3), in our cohort all steroid non-responders received a trial of rituximab, and none of them responded to this treatment. Newer treatment modalities targeting plasma cells, such as bortezomib and daratumumab, are gaining popularity in the treatment of multiple myeloma and have recently shown efficacy in treating AIC. Bortezomib, a 26S proteasome inhibitor, decreases the production of IgG antibodies by eliminating plasma cells and has been reported to effectively treat post-HSCT AIHA (14, 15). In our cohort, two patients received bortezomib, with no response. Daratumumab, an anti-CD38 monoclonal antibody developed to target malignant plasma cells in multiple myeloma, was also reported to effectively treat refractory post-HSCT AIC in several recent case reports (16). In our cohort, four patients received daratumumab, and in one of the patients (patient #4), a dramatic recovery from a refractory AIHA following the failure of several other treatments was observed (17). However, in three other patients in our cohort, there was no response to daratumumab, suggesting multifactorial pathogenesis of this complication and refractoriness to treatment in some cases.

Thrombopoietin receptor agonists such as eltrombopag and romiplostim, are used for the treatment of persistent/chronic ITP and have been used for post-HSCT thrombocytopenia in recent years with promising outcomes (18). Three of our patients were treated with eltrombopag, and in two of them, there was a good response and improvement in platelet counts following treatment.

Other third-line treatment options we have tried in refractory cases included rapamycin, an mTOR inhibitor that has shown efficacy in refractory cytopenias (19), and abatacept, a fusion protein that inhibits T-cell activation by binding to CD80/CD86 on antigen-presenting cells thus blocking the CD28 interaction with T-cells (20). Both have failed to show significant responses in our patients. Plasmapheresis, done for direct removal of circulating antibodies, was tried in one refractory case (patient #6), who eventually responded to multiple treatments and completely recovered.

As a last resort, a second transplant may reset the whole immune system, aiming to restart the process of immune reconstitution, at the price of significant treatment-related morbidity and mortality. In our cohort, two patients ended up undergoing a repeat HSCT due to prolonged AIC with a constant need for blood products and refractoriness to other treatments (patients #2,5). One is alive and well, with normal counts (patient #2). The other, who rejected his 1^st^ transplant and developed refractory AIC after his 2^nd^ HSCT, died three weeks after his 3^rd^ HSCT from sepsis and multi-organ failure (patient #5).

Novel treatment modalities for the more refractory cases are direly needed. Strategies to increase peripheral tolerance by enhancing the donor Treg engraftment may be the key (21), as illustrated in patients with IPEX (immune dysregulation, poly-endocrinopathy, enteropathy, x-linked) syndrome. The phenotype of these patients is caused by mutations in FoxP3, leading to a lack of functional Tregs. HSCT, specifically donor Treg engraftment, has been shown to cure these patients and demonstrates the impact of functioning Tregs on immune regulation (22, 23).

## Conclusion

In conclusion, post-HSCT AIC presents with a variable presentation and a wide spectrum of severity. Some of these cases are prolonged and refractory, even requiring a repeated HSCT. In the rare group of MIOP patients, AIC is relatively prevalent, possibly due to the difficult engraftment and high incidence of mixed chimerism in these patients. There is a dire need for novel treatment modalities for better management of the more severe and refractory cases.

## Data Availability Statement

The raw data supporting the conclusions of this article will be made available by the authors, without undue reservation.

## Ethics Statement

The studies involving human participants were reviewed and approved by Hadassah Medical Centers’ Ethics Committee. Written informed consent from the participants’ legal guardian/next of kin was not required to participate in this study in accordance with the national legislation and the institutional requirements.

## Author Contributions

EE-O and PS conceptualized and drafted the manuscript; YS, AN, IZ, and BS reviewed and edited the manuscript. All authors contributed to manuscript revision, read, and approved the submitted version

## Conflict of Interest

The authors declare that the research was conducted in the absence of any commercial or financial relationships that could be construed as a potential conflict of interest.

## Publisher’s Note

All claims expressed in this article are solely those of the authors and do not necessarily represent those of their affiliated organizations, or those of the publisher, the editors and the reviewers. Any product that may be evaluated in this article, or claim that may be made by its manufacturer, is not guaranteed or endorsed by the publisher.
